# Accuracy of Pediatric Risk of Mortality (PRISM) III Score in Predicting Mortality Outcomes in a Pediatric Intensive Care Unit in Karachi

**DOI:** 10.7759/cureus.7489

**Published:** 2020-03-31

**Authors:** Sadiq Mirza, Laraib Malik, Jawad Ahmed, Farheen Malik, Hassaan Sadiq, Sanower Ali, Sina Aziz

**Affiliations:** 1 Pediatric Critical Care, The Indus Hospital, Karachi, PAK; 2 Pediatrics, Abbasi Shaheed Hospital, Karachi, PAK; 3 Internal Medicine, Dow University of Health Sciences, Karachi, PAK; 4 Surgery, Civil Hospital, Dow University of Health Sciences, Karachi, PAK; 5 Community Health Science, Jinnah Dental and Medical College, Karachi, PAK; 6 Pediatrics, Karachi Medical and Dental College, Karachi, PAK

**Keywords:** pediatrics, paediatrics, pediatric mortality, prism, pediatric intensive care unit, picu, emergency medicine, mechanical ventilation, inotropic drugs

## Abstract

Background

With the advancements in medicine and increasing access to modern technology, pediatric intensive care units (PICU) are becoming a vital part of any health care setting. PICUs play a key role in saving the life of young patients. Various scales have been designed by researchers to aid in predicting the mortality of a patient admitted in PICU. Pediatric Risk of Mortality (PRISM) and Pediatric Index of Mortality (PIM) are among the most commonly used scales. Calculating the risk of mortality enables the physicians to classify the patients and helps in identifying which patients require more urgent care and resources.

Methods

A hospital-based prospective study was carried out at PICU in a tertiary care hospital in Karachi from December 2017 to June 2019. All patients between the age of one month and 12 years were included in our study after informed consent from parents/guardians. A standard questionnaire was used and the PRISM III score was calculated at 24 hours of admission. All necessary investigations were carried out, and all statistical analyses were carried out using SPSS v.23 (IBM, Armonk, NY).

Results

A total of 407 patients were included in our study with the majority being males (54.5%). The mean age was 27±33 months. The mean duration of stay of patients in PICU was 80.15±36.58 hours. The mortality rate in our study was 37.35 % (n=152). The need for mechanical ventilation, use of inotropic drugs, higher temperatures, and low Glasgow Coma Scale scores were associated with poor survival. It was noted that as the PRISM III score increased, the mortality rate also increased. In our study, we found that PRISM III had good predictive power in our population. The area under the curve was 0.903±0.016 (p<0.001, 95% confidence interval: 0.872-0.934).

Conclusions

PRISM III score showed excellent accuracy and predictive ability in our population. There was no significant difference in observed and expected mortality rates in our study. In a resource-limited setting, the prediction models highlight the cases where more medical attention is required and also enable the physicians to assess the prognosis of the patient so adequate measures can be taken beforehand.

## Introduction

The pediatric intensive care units (PICUs) have drastically improved in Pakistan in the last decade. With the availability of specialized life supporting machines and well-trained staff, intensive care units (ICU) are playing a key role in combating life-threatening conditions and illnesses. PICUs work with the fundamental purpose of reducing mortality by intensively monitoring and treating patients with potentially fatal physiological dysfunctions. Estimating the risk of mortality in ICU allows the physicians to assess the prognosis of the patient, plan therapies, and aid in evaluating the performance and resource utilization in an ICU [1-3].

The prediction of mortality risk by pediatricians is highly subjective (qualitative); therefore, there is a need for a scoring system (quantitative) for patients admitted to PICU. Clinical scoring systems have become a vital instrument in ICU. Index of Physiological Stability score (1984) consisting of 34 variables was one of the initial scoring systems used in pediatrics [4]. In 1988, Pollack et al. designed Pediatric Risk of Mortality (PRISM) score for prediction of mortality in PICU. It consisted of 14 variables [5]. PRISM was later modified to PRISM III with an addition of three variables by Pollack in 1996. PRISM III (17 variables) was tested among 11,165 patients in 32 PICUs across the USA and yielded better results than PRISM in predicting mortality [6]. Prediction of mortality can be assessed using 12 hours (PRISM III-12) or 24 hours (PRISM III-24) data. PRISM III-24 is more accurate for individual patient’s mortality prediction, whereas PRISM III-12 is primarily used in qualitative studies [6]. In Europe, the Pediatric Index of Mortality (PIM) consisting of eight variables was put forward by Shann et al. and it was done immediately after admission of the patient [7].

One of the drawbacks of mortality predicting models is that they are population sensitive. A pilot study is necessary before the initiation of their use in any PICU. The PRISM III has been studied with successful results in various PICUs; however, an Italian study carried out in 26 PICUs did not support the predictive power of PRISM III [8-12]. There is only limited data related to the application of PRISM III in tertiary hospitals across Pakistan. This study aimed to evaluate the suitability of PRISM III for our population and to determine the accuracy of the PRISM III score in predicting the mortality outcome of patients admitted in PICU of a tertiary care hospital in Karachi. 

## Materials and methods

Study design and duration

A prospective hospital-based study was carried out at PICU of Abbasi Shaheed Hospital (ASH) Karachi from December 2017 to June 2019. PICU at ASH comprises of five beds and admits children from one month to 12 years of age.

Study population and inclusion criteria

The data were collected using a consecutive sampling technique. Critically ill pediatric patients between one month and 12 years of age who were admitted directly to the PICU of our hospital or referred from some other hospitals due to any medical or surgical emergencies were included in our study for one and a half year. Informed consent from the patient’s parents/guardian was obtained prior to inclusion in the study.

Exclusion criteria

Exclusion criteria were defined as follows: (1) all the patients that were ≤1 month and >12 years of age, (2) had an underlying malignancy or congenital malformation, and (3) left against medical advice within 24 hours of admission.

Ethical approval

Ethical approval was obtained from the Ethical and Scientific Review Committee, Karachi Medical & Dental College (Ref: 007/17, dated April 27, 2017). 

Study tool

On admission and after appropriate consent, a standard PRISM III-24 questionnaire was used for the study patients meeting the inclusion criteria. As per standard medical form, demographic data such as gender, age, diagnosis, nature of outcome (survival/non-survival), and duration of stay in PICU, a requirement of ventilator support, vital monitoring (heart rate, temperature, blood pressure, Glasgow Coma Scale [GCS] score, and pupillary reactions), and lab parameters were filled in for each patient. Laboratory investigations for arterial blood gases parameters, pH, and levels of glucose, potassium, creatinine, blood urea nitrogen, and liver function tests were carried out. A complete history, thorough physical examination, and appropriate laboratory investigations were carried out for each patient.

Statistical analysis

All the data were pooled into Statistical Package for Social Science (SPSS) software version 23.0 (IBM, Armonk, NY) and all the analyses were carried out through it. Continuous variables such as weight, age, and length of PICU stay were reported as means or medians with standard deviations. T-test and chi-square tests were used to find any relation between different variables. The receiver operating characteristics (ROC) curve was plotted to find the sensitivity and specificity of PRISM III. The Hosmer and Lemeshow goodness-of-fit chi-square test was carried out to see the goodness of the predictive model.

Data were collected, and the PRISM III score was calculated within 24 hours of admission at PICU. The PRISM III score evaluation was done as per the recommendation of Pollack et al. [6]. The outcome was calculated from the total score achieved by each patient as survivors versus non-survivor.

## Results

A total of consecutive 572 patients were approached for the study, and of these 407 (54.5% boys/45.5% girls) were included in our study. The rest of the patients were not analyzed based on exclusion criteria or insufficient data. The mean age and weight of the participants were 27±33 months and 10.20±7.94 kg, respectively. The mean duration of PICU stay was 80.15±36.58 hours. Study characteristics and different presenting diseases are displayed in Table 1 and Table 2, respectively.

**Table 1:** Demographic profile of the study. P-value shows the association of the variables with mortality outcome. PICU, pediatric intensive care unit; GCS, Glasgow Coma Scale.

Variables	N (%)	P-value
Total patients	407	
Gender		0.067
Males	222 (54.5)	
Females	185 (45.5)	
Age (months)		
> 1 –12	239 (58.72)	
> 12–60	108 (26.54)	
> 60–120	60 (14.74)	
> 120	0 (0)	
Mean age (months)	27±33	0.075
Median age (months)	9±33	0.075
Mean weight (kg)	10.20±7.94	0.342
Mean PICU stay (hours)	80.15±36.58	0.087
Temperature <33 or <40°C	7 (1.7)	0.008
GCS score		<0.0001
More than 8	322 (79.1)	
Less than 8	85 (20.9)	
Mechanical ventilation		0.009
Yes	143 (35.2)	
No	264 (64.9)	
Inotropic drugs		0.005
Yes	160 (39.4)	
No	247 (60.6)	
Outcome		
Survived	255 (62.65)	
Expired	152 (37.4)	

**Table 2:** Presenting diseases and the distribution of mortality rates. SCD, sickle cell disease; TB, tuberculosis; AFP, acute flaccid paralysis; CLD, chronic liver disease; CF, cystic fibrosis *Each case had an incidence of n=1.

Diagnosis	N (%)	Mortality (%)
Acute exacerbation of asthma	24 (5.9)	3 (12.5)
Bronchiolitis	72 (17.7)	11 (15.3)
Encephalitis (bacterial or fungal)	29 (7.1)	23 9 (79.3)
Enteric fever with complications	3 (0.7)	1 (33.3)
Guillain-Barré syndrome	6 (1.6)	4 (66.7)
Measles with complications (including encephalitis or pneumonia)	42 (10.3)	27 (64.3)
Meningitis	101 (24.8)	47 (46.5)
Pneumonia	83 (20.4)	14 (16.9)
Poisoning	14 (3.4)	6 (42.9)
Post measles encephalitis	8 (2.0)	6 (75)
Sepsis	16 (3.9)	7 (43.8)
SCD, status epileptics, TB with effusion, thalassemia, AFP (polio), CLD, CF, pneumothorax, and head trauma*	9 (2.21)	3 (33.3)
Total	407	152

Mortality and variables

Out of the 407 patients, 152 died (non-survivors) in PICU and 255 survived. The overall mortality rate was 37.35%. No significant association was found between gender, age, and length of PICU stay of the patient with the mortality outcome (p=0.067, p=0.075, and p=0.087 respectively). Need for mechanical ventilation (p=0.009), use of inotropic drugs (p=0.005), higher temperature (p=0.008), and low GCS (p<0.0001) were associated with poor outcome. A significant association was noted between mortality outcome with systolic blood pressure (p<0.0001) and pupillary reflex (p<0.0001), while no association was observed for heart rate (p=0.432) and pH (p=0.79).

PRISM III score and mortality

**Figure 1:**
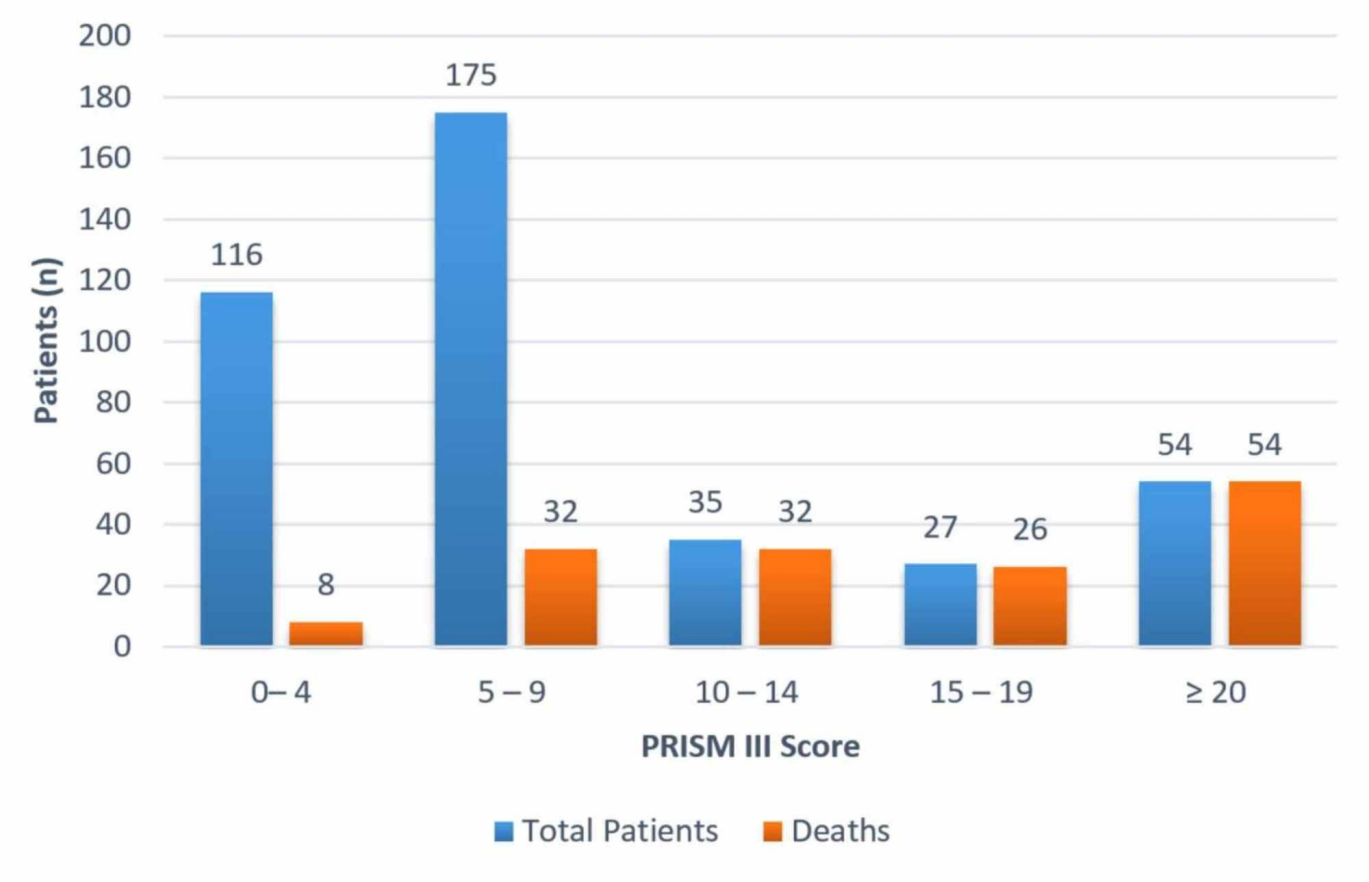
Distribution of mortality according to PRISM III score. PRISM, Pediatric Risk of Mortality.

A significant association was found between the PRISM III score and mortality outcome (p<0.001). The mean PRISM III score was 8.32±7.07. A total of 291 patients had a score of <10 and the mortality rate among them was 13.75%. Among 62 patients who had a score of 10 to 19, 93.54% of patients expired and only 6.46% of patients survived. The mortality rate was 100% in patients who had a score of >20. As the PRISM III score increased, the rate of mortality also increased. A more detailed distribution of PRISM III score with mortality rate is shown in Figure 1.

Goodness of prediction of PRISM III

The predicted mortality rate by the PRISM III scores correlated well with the actual observed mortality rate as shown in Table 3. The Hosmer and Lemeshow goodness-of-fit chi-square test was used to appreciate the goodness of prediction. The result showed that the expected mortality rate was 37.7% and the observed mortality rate in our study was 37.4%. No significant difference was seen between the expected and observed mortality rate (p=0.631). A p-value of >0.05 is considered good suitability of the test.

**Table 3:** Goodness of the predictive model by the Hosmer and Lemeshow chi-square test. PRISM, Pediatric Risk of Mortality.

PRISM III score	Total	Survival		Expired	
		Observed	Expected	Observed	Expected
0–4	116	108	107.82	8	8.09
5–9	175	143	142.30	32	32.77
10–14	35	03	3.11	32	31.86
15–19	27	01	0.83	26	24.91
≥20	54	00	0.05	54	55.69
Total	407	255	254.11	152	153.32

Accuracy of prediction of PRISM III

**Figure 2:**
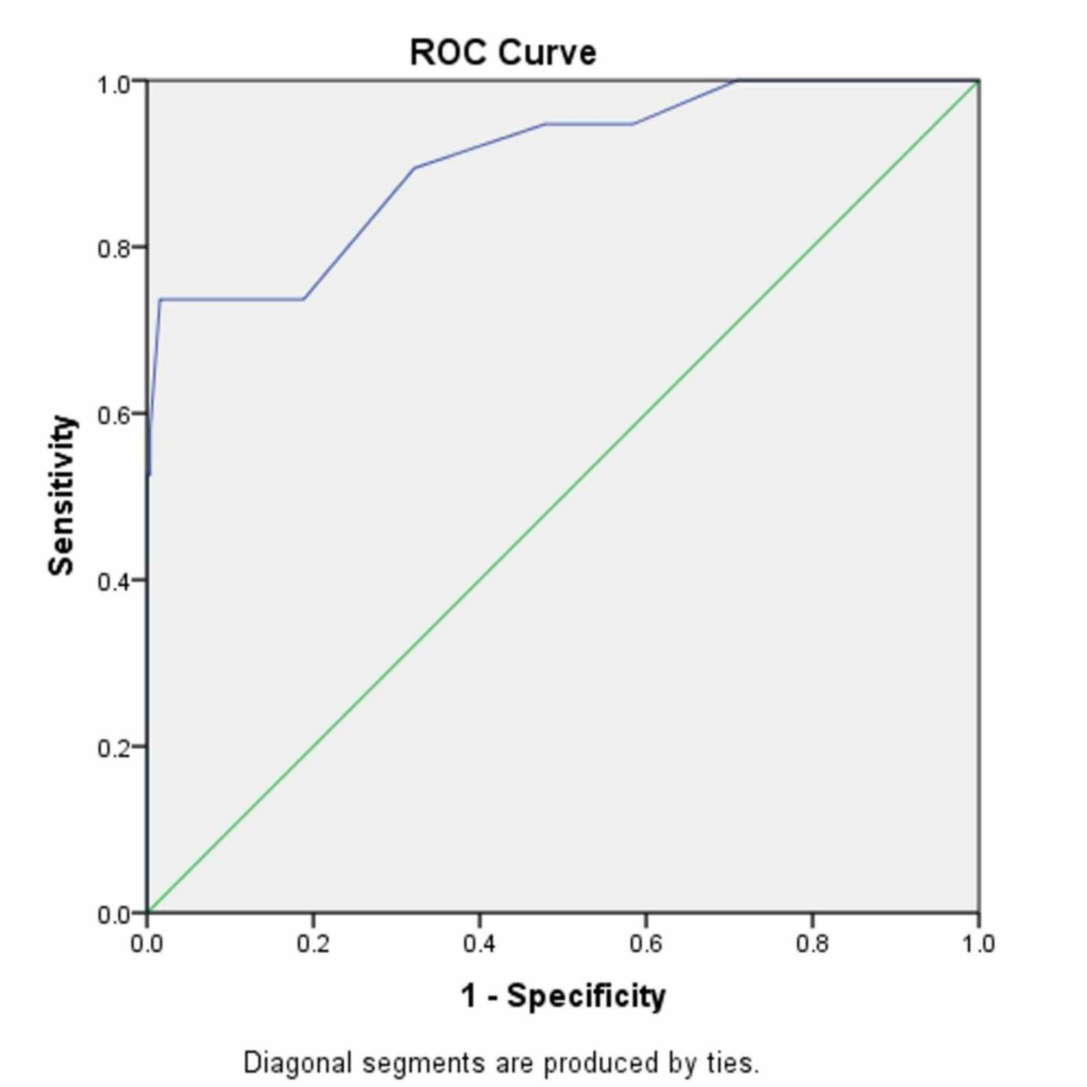
ROC curve for PRISM III score. Area under the curve is 0.903±0.016 (p<0.001, 95% CI: 0.872-0.934). ROC, receiver operating characteristics; PRISM, Pediatric Risk of Mortality; CI, confidence interval.

PRISM III scores offered a good discriminative power in our center with 0.903 (95% CI) area under the ROC curve (Figure 2). The area under the curve is a measure of the overall accuracy of the model as well as its ability to predict mortality. The closer the area under the ROC curve is to 1.0, the more accurate the model. Taking 10 as cut-off point, the sensitivity and specificity of PRISM III model in our population were 73.7% and 98.4%, respectively.

## Discussion

Prediction models of mortality provide great insight to health care administrators regarding the prognosis of the patient and may greatly benefit the decision process as well as the outcome of the patient.

A variety of models exist for the prediction of mortality in patients of PICU, including the Pediatric Risk of Mortality (PRISM, PRISM III) and the Pediatric Index of Mortality (PIM and PIM 2) [5,7,13]. Regardless, globally the PRISM III score is used more frequently and we decided to utilize it as well. Although these prediction models are an asset to intensive care, it is essential to validate them before applying in an environment significantly dissimilar from where they were initially developed. The purpose of this study was to use a critical illness scoring system like PRISM-III to assess the mortality risk and its validity in predicting the outcome of patients admitted to PICU at a tertiary care hospital with resource-limited settings.

The findings from this study elucidate that the mortality rate predicted by the PRISM III score correlated well with the actual observed mortality rate, thus providing an accurate estimate of the prognosis and outcome of the patients admitted in PICU.

In this study, the observed mortality rate was 37.35%. This mortality is significantly higher when compared to comparable studies carried out in developing countries [9,14,15]. This highlights the need for better resource allocations and practices in the PICU in our country. A directly proportional relationship was noted between the PRISM III score and the mortality, that is as the PRISM III score increases, mortality rate also increases. This finding was similar to researches carried out in India, Hong Kong, and the UK [9,16,17]. In most of the studies done previously, the primary illness is usually due to trauma, hereditary disease, or malignancies. Conversely, in our study population, communicable and infectious diseases like meningitis (24.8%) and pneumonia (20.4%) were more common. This was concordant with a previous study done in Pakistan [15].

The distribution of gender in our research was analogous to previous studies carried out in the nearby region, but no significant association was seen between gender and mortality [15,18]. This finding was comparable to previous work done in Brazil and Nepal [14,19]. In contrast, Aragao et al. discovered in his study population that males had a greater risk of mortality than females [20]. Our patients were considerably younger (median age, nine months) in comparison to the study done originally by Pollak et al. to validate PRISM, which was 33 months [5]. We further observed that the use of inotropic drugs (p=0.005) and mechanical ventilation (p=0.009) were considered risk factors for mortality, which was consistent with other studies where procedures like these were considered high risk for the pediatric patients [14,19].

PRISM III-24 contains 17 physiological variables. These variables include but are not limited to systolic blood pressure, mental status, heart rate, and glucose. A significant association between these physiologic variables and mortality outcome has been uneven across the literature as elucidated by Table 4. This table compares different variables in our study with other research studies reported worldwide.

**Table 4:** Comparision of the significance of physiologic variables among different studies. GCS, Glasgow Coma Scale; BP, blood pressure.

Study name	Location	Mortality outcome (%)	GCS score	Systolic BP	Heart rate	Pupillary reflexes	pH
Our study	Pakistan	37.35	Significant	Significant	Non-significant	Significant	Non-significant
Varma et al [9]	India	14.8	Significant	Significant	Non-significant	Significant	Significant
Ana Lilia et al [8]	Mexico	24.7	Non-significant	Non-significant	Non-significant	Significant	Significant
Pollack et al [6]	USA	2.2 to 16.4	Significant	Significant	Non-significant	Significant	Non-significant

Scoring models suggest that a discriminatory power of 0.90 and above is deemed excellent, 0.80 to 0.89 to be good, and 0.70 to 0.79 to be fairly suitable [21,22]. Furthermore, the predictive ability of a model can be assessed by estimating how close the ROC curve is to 1.0. The present study demonstrates that PRISM III acts as an excellent discriminative tool (0.903 area under the ROC curve). Previous studies conducted in Pakistan have yielded similar results to ours. Siddique et al. and Quereshi et al. demonstrated PRISM to have a good discriminative and predictive ability with an area under the ROC curve of 0.885 and 0.78, respectively [15,18]. Choi et al. reported that the area under the ROC curve for PRISM was 0.910 and was an excellent predictor of mortality in intensive care settings [16]. Studies that were carried out in Iran and India also observed a good discriminative ability of PRISM with an area under the ROC curve to be greater than 0.8 and 0.86, respectively [9,23].

Our study incorporated a variety of patients with different illnesses which shows that the PRISM III was suitable for varying morbidities. The other strength of our study was a large sample size which made our results more robust. However, our study was limited by the fact that it was a single-center study. Caution has to be taken before generalizing the results for the population of various areas where resources are more limited. A wider study needs to be conducted that encompasses hospitals from different cities of Pakistan.

## Conclusions

PRISM III was found to be an excellent predictor of mortality in our population. Factors such as mechanical ventilation, inotropic drugs use, and low GCS scores were associated with poor outcomes. More than half of our presenting population was younger than one year, and infectious causes were the main reason for admission in most of the cases. Using the prediction models, physicians can assess the survival chances of the patient. In settings where there is a shortage of medicines and staff, such models enable physicians to decide how and where to direct their limited resources. 
